# Supplementary material for: TNFα Rescues Dendritic Cell Development in Hematopoietic Stem and Progenitor Cells Lacking C/EBPα
Source: Cells. 2020 May 15;9(5):1223. doi: 10.3390/cells9051223 (PMC7291045; doi:10.3390/cells9051223)
Supplement: Supplementary file 1 [file cells-09-01223-s001.pdf]

Supplementary Table S1: Reagents, Antibodies and resources used in the study

Reagent/Resource	Supplier	Identifier
**Anitbodies**		
Biotin Mouse Lineage Panel	BD bioscience	# 559971
PE-Cy7 conjugated Ly6 - A/E (sca1) (Clone D7)	eBiosceince	# 25-5981-82
APC conjugated CD117(Ckit) (Clone 2B8)	BD pharmigen	# 553356
7-AAD	BD pharmigen	# 559925
APC conjugated CD11c (Clone HL3)	BD pharmigen	# 550261
APC-Cy7 conjugated MHC II (Clone M5/114.15.2)	Biolegend	# 107627
PE-Cy7 conjugated CD117(Ckit) (Clone 2B8)	eBioscience	# 25-1171-82
APC conjugated FLT3 (Clone A2F10)	Biolegend	# 135310
PE-Conjugated CD115 (Clone AFS98)	eBioscience	# 12-1152-82
**Arrays**		
GeneChip™ Mouse Gene 2.0 ST Array	Affymetrix	# 902119
Bio-Plex™Cytokine Assay (Custom Analyte array)	Bio Rad Laboratories	See supp table S3
**Chemicals, Commercial assays and Recombinant Proteins**		
pIpC (Polyinosinic-polycytidylic acid sodium salt)	Sigma Aldrich	# P0913
Pharm Lyse 10x - Lysing Buffer	BD bioscience	# 555899
Mouse Hematopoetic Progenitor Cell Enrichment Set (Lineage Depletion Cocktail)	BD Biosciences	# 558451
IMag Buffer	BD Biosciences	# 552362
Recombinant Human Flt3-Ligand	Peprotech	# 300-19
Recombinant Murine TNF-α	Peprotech	# 315 - 01A
RNeasy MicroKit (50)	Qiagen	# 74004
High-Capacity cDNA Reverse Transcription Kit	Applied Biosystems	# 4368814
TaqMan assays for RT-qPCR	Applied biosystems	# 4453320
**Experimental Models**		
Mx1-Cre Cebpa F/F (Cebpa inducible Knockout mice)	Zhang DE et al. , 1997	
Cebpa-Cre EYFP (Lineage flourescent tracer mice)	Wölfler A et al. , 2010	
**Softwares**		
Kaluza Analysis Software	Beckman Coulter	
Gene Expression Console v1.1	Affymetrix	
BioVenn - Venn Diagrams	Hulsen T et al. , 2008	
Network Analyst 3.0	Xia J et al. , 2013, Zhou G et al. , 2019	
Prism 6	GraphPad	
**Others**		
BD Facs LSR II	BD Bioscience	
LightCycler 480 instrument	Roche	
BD FACS Aria	BD Bioscience	

Supplementary Table S2: TaqMan Probes used in the study

Gene Name	Assay ID
Cebpa	Mm00514283_s1
Gapdh	Mm99999915_g1
β-Actin	Mm02619580_g1
Cx3Cr1	Mm02620111_s1

Supplemental Table S3: Cytokines analysed using BioPlex™ assay

CCL2/JE/MCP-1	G-CSF
CCL3/MIP-1 alpha	GM-CSF
CCL4/MIP-1 beta	IFN-gamma
CCL5/RANTES	IL-1 alpha
CCL20/MIP-3 alpha	IL-1 beta
CXCL1/KC	IL-4
CXCL2/MIP-2	M-CSF
CXCL10/IP-10/CRG-2	TNF-alpha
CXCL12/SDF-1 alpha	

Cebpa q-PCR

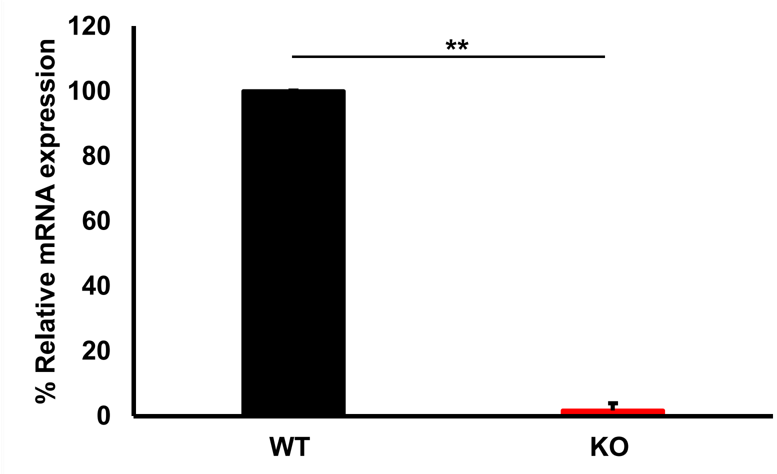

S**upplementary Figure S1**: **qPCR analysis of *Cebpa* expression in HSPCs.** qPCR analysis of HSPCs isolated from $Mx1^{Cre}/Cebpa^{F/F}$ (KO) mice and their $Cebpa^{F/F}$ (WT) littermates reveals almost absent Cebpa expression in KO HSPCs. (n=3 mice, ** $p < 0.01$).

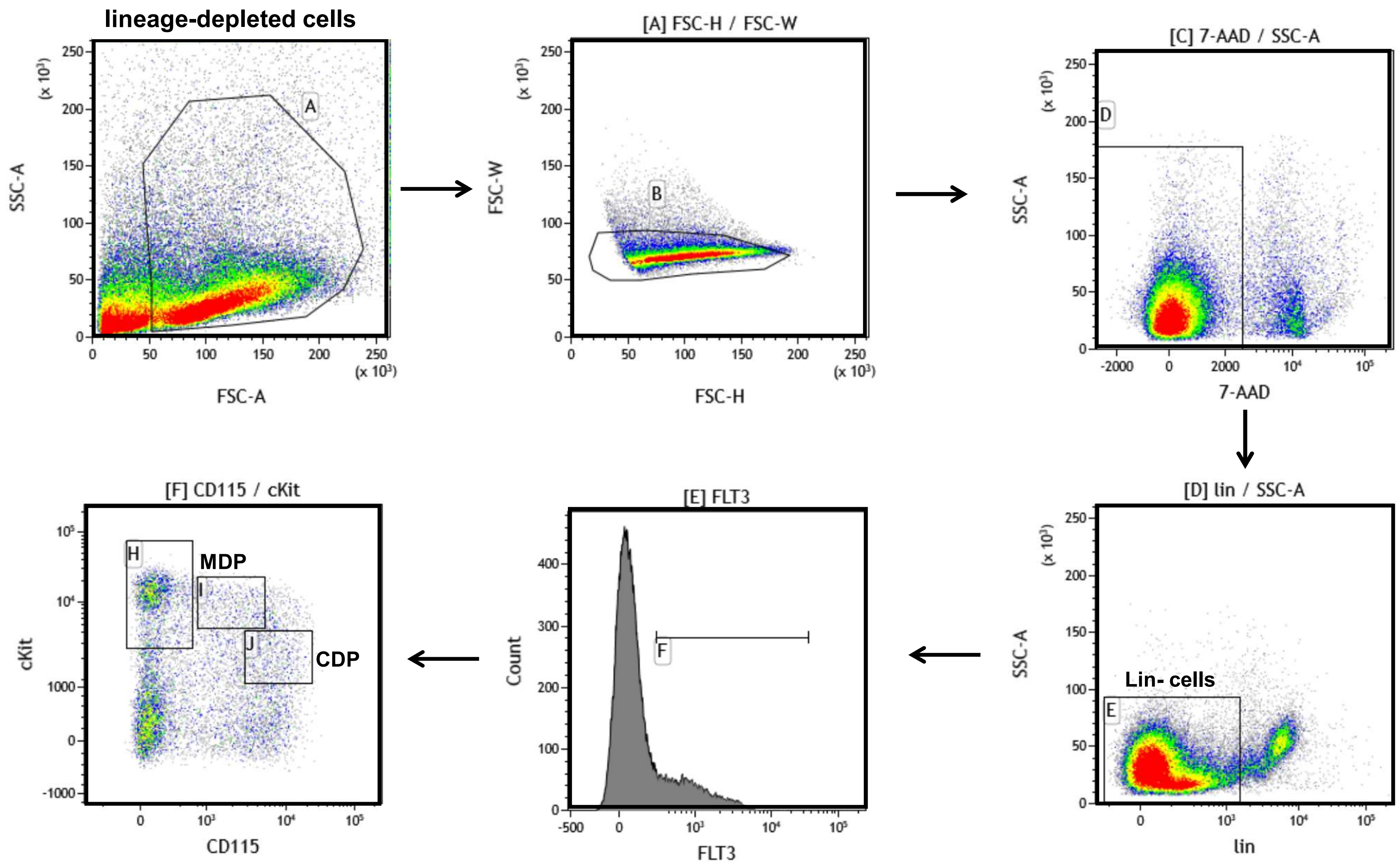

Supplementary Figure S2: Gating strategy of MDPs and CDPs of bone marrow cells after lineage depletion.

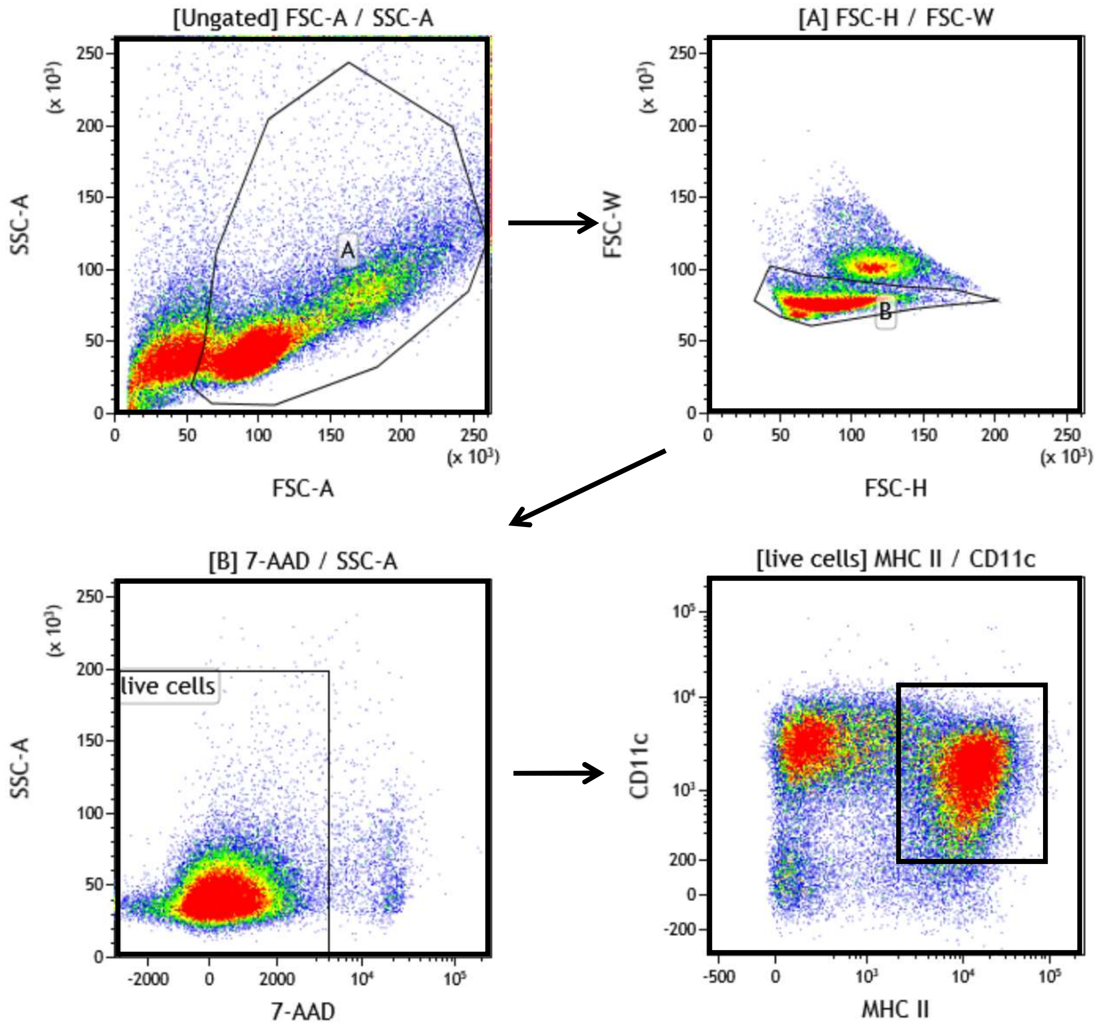

Supplementary Figure S3: **Gating strategy of DCs after in vitro generation using FLT3L stimulation for 8 days**

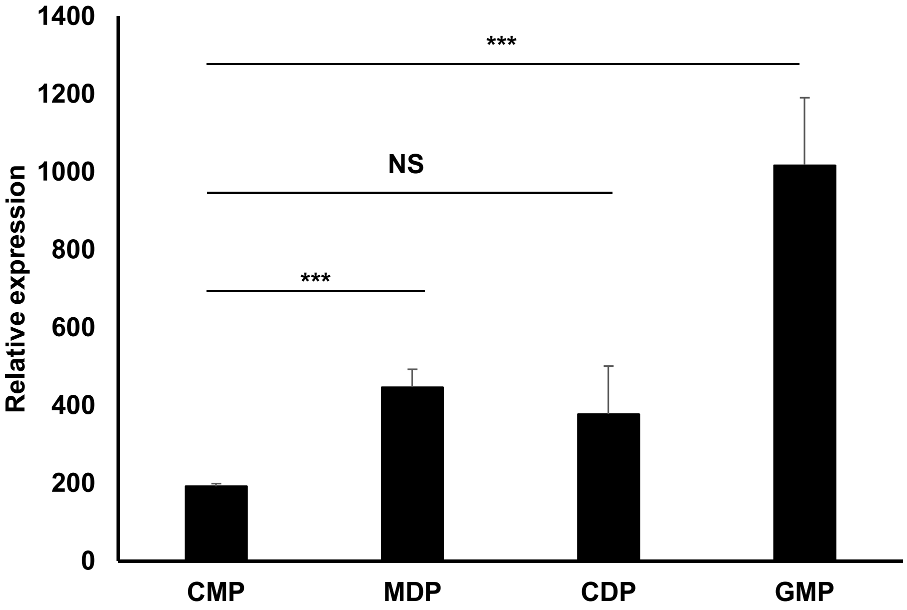

Supplementary Figure S4: **Analysis of Cebpa mRNA expression in DC progenitors.** Gene expression data from Miller et al., Nature Immunology 2012 (GEO: GSE15907) including CMPs, MDPs, CDPs and GMPs were analyzed. We observed an increased Cebpa expression in MDPs and CDPs as compared to CMPs. As expected from the results of the Cebpa Cre EYFP mice, the highest Cebpa expression was observed in GMPs (*** $p < 0.001$; NS denotes not significant).

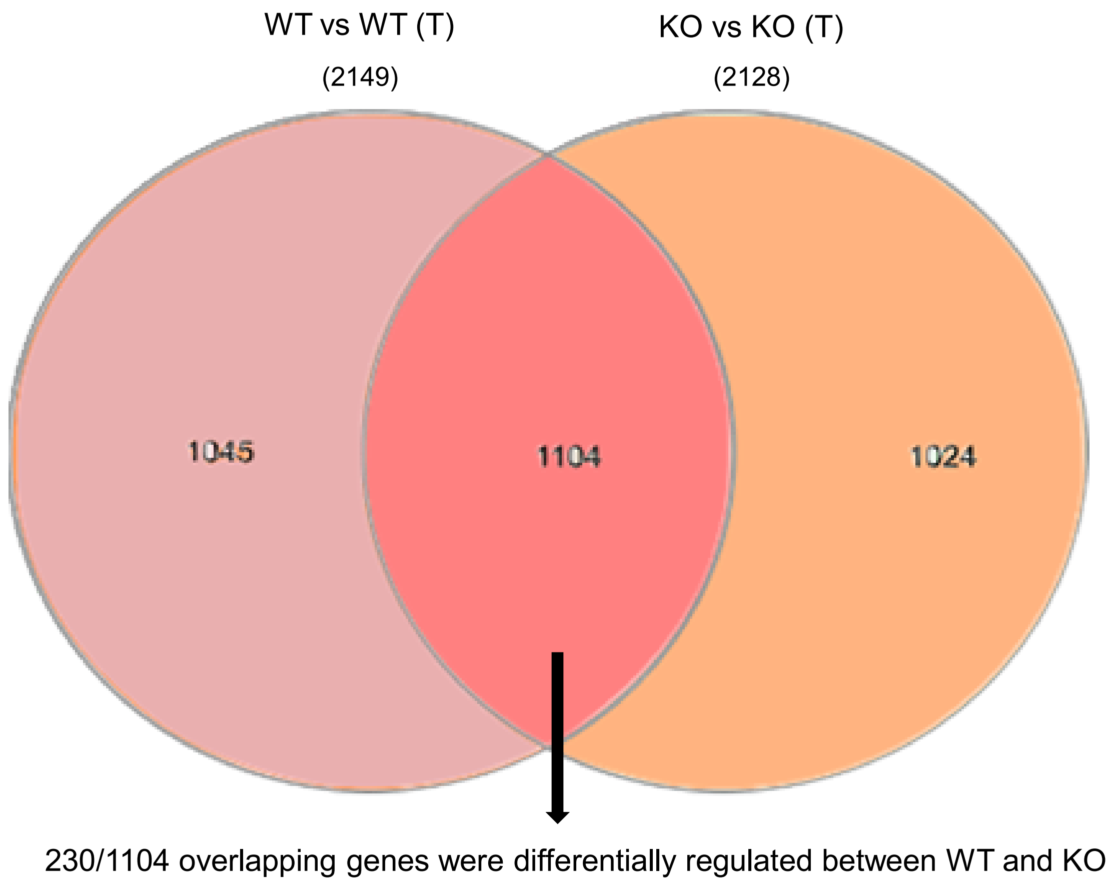

S**upplementary Figure S5**: **Venn diagram summarizing microarray analysis.** Venn diagram showing the overlap between differentially regulated genes during DC development, in the presence and absence of Cebpa. All gene included in the for analysis show >1.5 fold difference with a FDR5% corrected significance < 0.05.

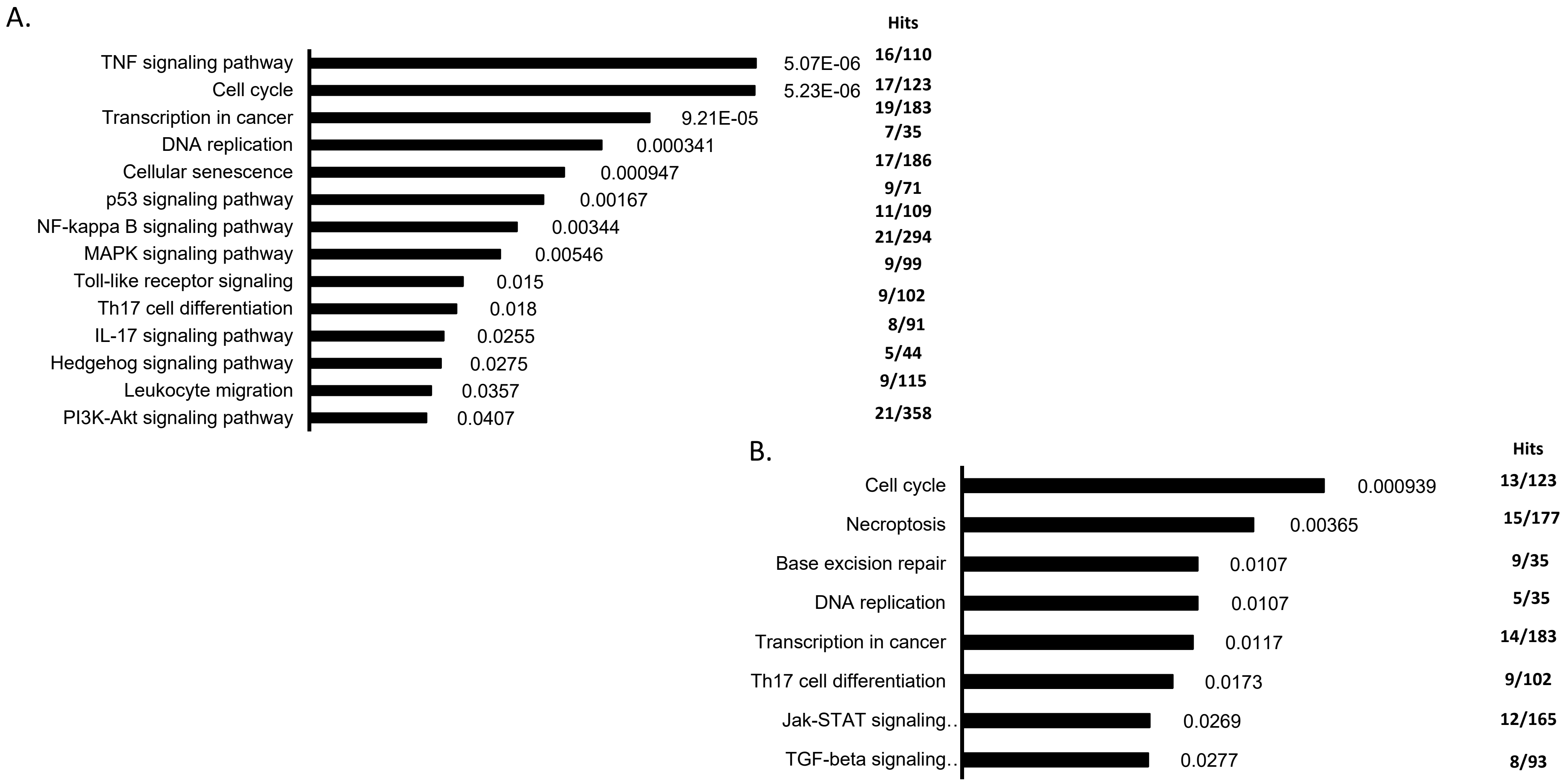

Supplementary Figure S6: Pathway analysis of additional gene lists. Pathway analysis of gene lists differentially regulated exclusively in A. WT vs WT(T) (1045) and B. KO vs KO (T) (1024).

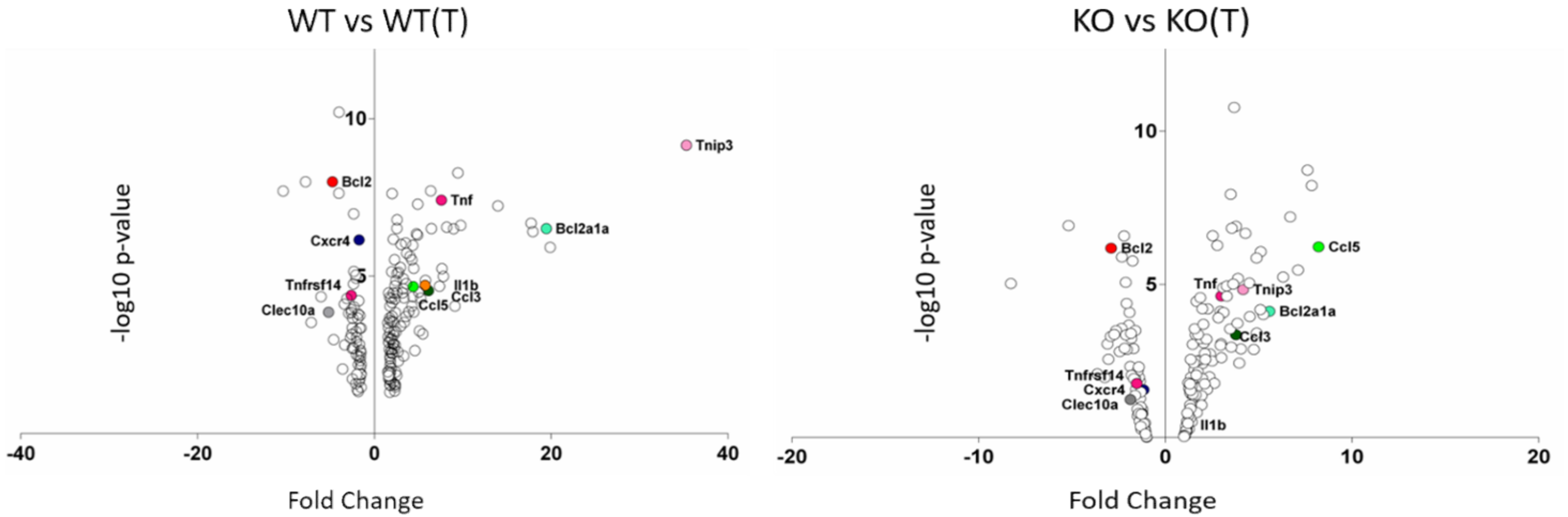

Supplementary Figure S7: Volcano plot based visualization. Representation of the most significantly regulated genes that were differentially regulated between the WT vs WT(T) and the KO vs KO(T)

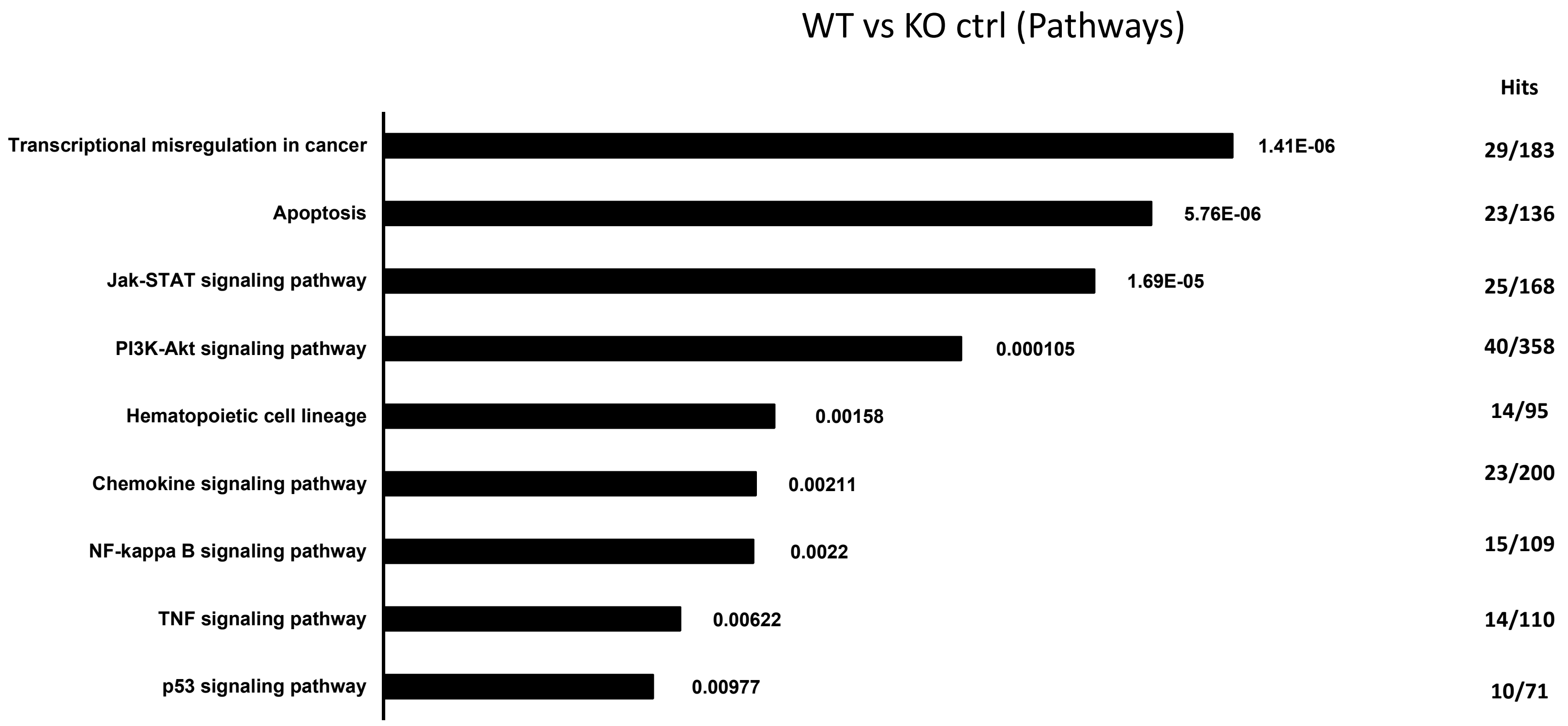

Supplementary Figure S8: Pathway analysis of differentially regulated genes in unstimulated WT vs. KO HSPCs. Pathway analysis of gene lists differentially regulated between unstimulated HSPCs of WT vs KO mice.

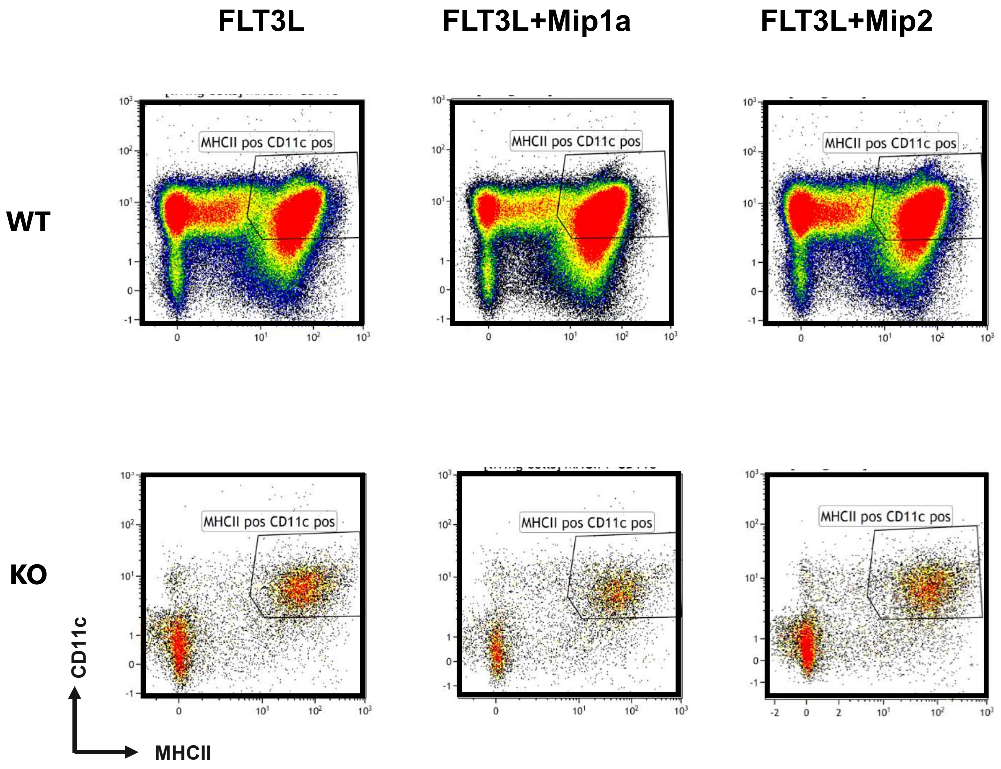

Supplementary Figure S9: Generation of CD11c⁺MHCII⁺ DCs in WT and KO HSPCs after stimulation with FLT3 +/- MIP1a or MIP2. Flow cytometric plots showing a lack of effect of MIP1a or MIP2 addition in KO HSPCs upon *in vitro* culture in presence of FLT3L**.**

KO HSPCs

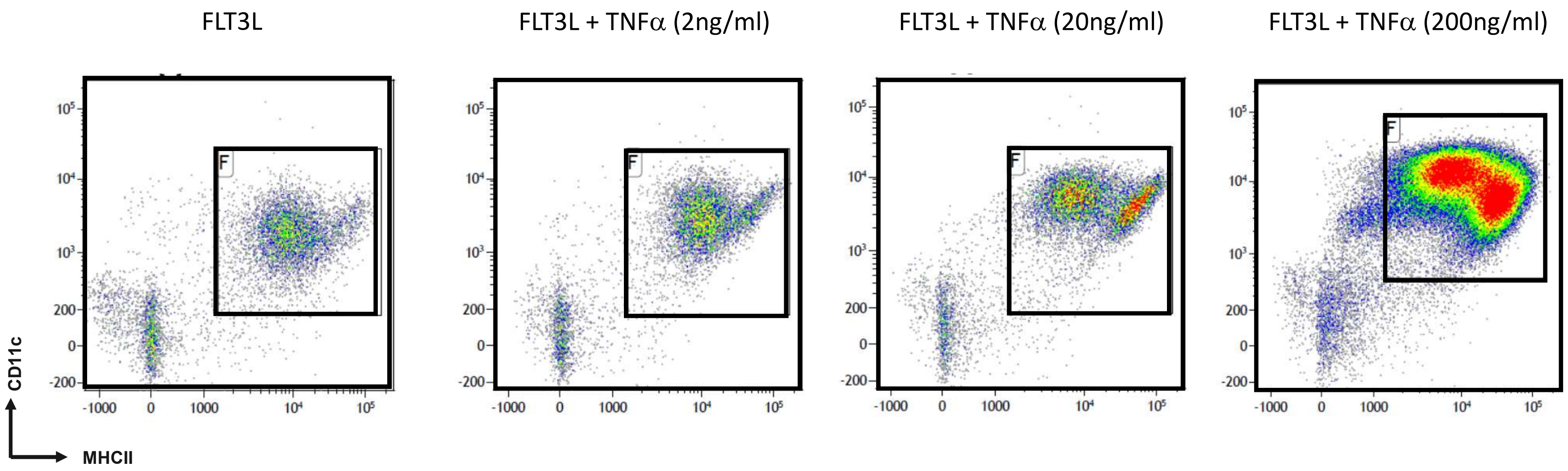

Supplementary Figure S10: Generation of CD11c⁺MHCII⁺ DCs in KO HSPCs after stimulation with FLT3L and increasing amounts of TNFα.

WT mice

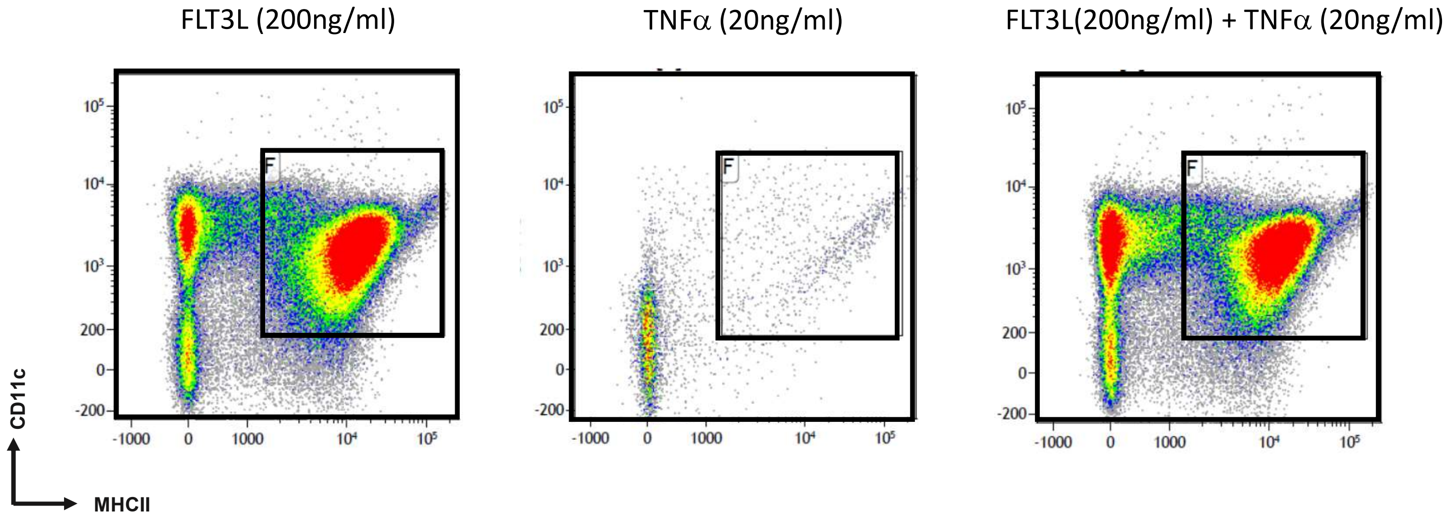

Supplementary Figure S11: Generation of CD11c$^+$MHCII$^+$ DCs in WT HSPCs after stimulation with FLT3L or TNFα alone or a combination of both cytokines.

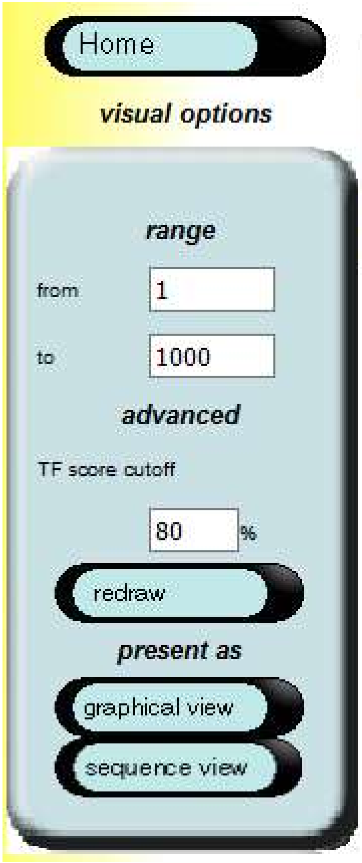

Transcription factor	Cx3Cr1				
	Sequence	From	To	Score	Strand
cEBP	TGGGGCAACGGG	252	263	7.094	+
cEBP	AGAGTTGTGATA	464	475	6.390	-
cEBP	GCTATTGGCTAA	952	963	6.995	-

Supplementary Figure S12: Transcription Factor Binding Site analysis. Predicted promoter sequence of Cx3Cr1 as predicted by Ensembl and confirmed by blasting against Promoter DB and EPD DB shows three possible cEBP bzip transcription factor binding sites on the Consite web based software, and confirmed with Promo 3.0
